# An efficient, non-invasive approach for *in-vivo* sampling of hair follicles: design and applications in monitoring DNA damage and aging

**DOI:** 10.18632/aging.203744

**Published:** 2021-12-06

**Authors:** Natalie Kudlova, Hanus Slavik, Pavlina Duskova, Tomas Furst, Josef Srovnal, Jiri Bartek, Martin Mistrik, Marian Hajduch

**Affiliations:** 1 Institute of Molecular and Translational Medicine, Faculty of Medicine and Dentistry, Palacky University and University Hospital Olomouc, Olomouc 779 00, Czech Republic; 2 Faculty of Science, Palacky University and University Hospital in Olomouc, Olomouc 779 00, Czech Republic; 3 Danish Cancer Society Research Center, Copenhagen DK-2100, Denmark; 4 Division of Genome Biology, Department of Medical Biochemistry and Biophysics, Science for Life Laboratory, Karolinska Institute, Stockholm 171 77, Sweden

**Keywords:** hair follicle cells, non-invasive sampling, irradiation, cellular senescence, DNA damage response

## Abstract

In accordance with the 3 Rs principle (to replace, reduce and refine) animal models in biomedical research, we have developed and applied a new approach for sampling and analyzing hair follicles in various experimental settings. This involves use of a convenient device for non-invasive collection of hair follicles and processing methods that provide sufficient amounts of biological material to replace stressful and painful biopsies. Moreover, the main components of hair follicles are live cells of epithelial origin, which are highly relevant for most types of malignant tumors, so they provide opportunities for studying aging-related pathologies including cancer. Here, we report the successful use of the method to obtain mouse hair follicular cells for genotyping, quantitative PCR, and quantitative immunofluorescence. We present proof of concept data demonstrating its utility for routine genotyping and monitoring changes in quality and expression levels of selected proteins in mice after gamma irradiation and during natural or experimentally induced aging. We also performed pilot translation of animal experiments to human hair follicles irradiated *ex vivo*. Our results highlight the value of hair follicles as biological material for convenient *in vivo* sampling and processing in both translational research and routine applications, with a broad range of ethical and logistic advantages over currently used biopsy-based approaches.

## INTRODUCTION

Hair follicles provide a very accessible source of biological material that can be non-invasively obtained from most mammals [1, 2]. Such material is particularly valuable for experiments with rodent models involving repeated sampling that currently require multiple invasive biopsies, which are relatively laborious and stressful for the animals [3]. Replacement of blood or skin tissue collection would also be convenient for specific examinations in human medicine [4–6]. This micro-organ structure also has other advantages in biomarker studies, including suitability for investigations of circadian rhythms [5, 7], and the presence of numerous cell types in a small area, which can be easily distinguished, such as keratinocytes, melanocytes, or perifollicular macrophages and mast cells [8–10]. However, the epithelial component of the follicles is the most promising for biomarker investigations because most neoplastic diseases have an epithelial origin.

Here, we present a proof of concept study involving use of a newly designed and constructed device allowing collection of large amounts of murine hairs with their hair roots containing follicular cells. We also demonstrate that DNA, RNA, and proteins in the collected hair follicle cells can be conveniently analyzed using standard cell and molecular biology methods such as genotyping, qPCR, and immunofluorescence analyses. The study focused on changes associated with the DNA damage response (DDR), especially double-strand breaks, and cellular senescence both in naturally aged tissue and induced using ionizing radiation (IR).

## RESULTS

### Design of the follicular cells’ collector

**Figure 1:**
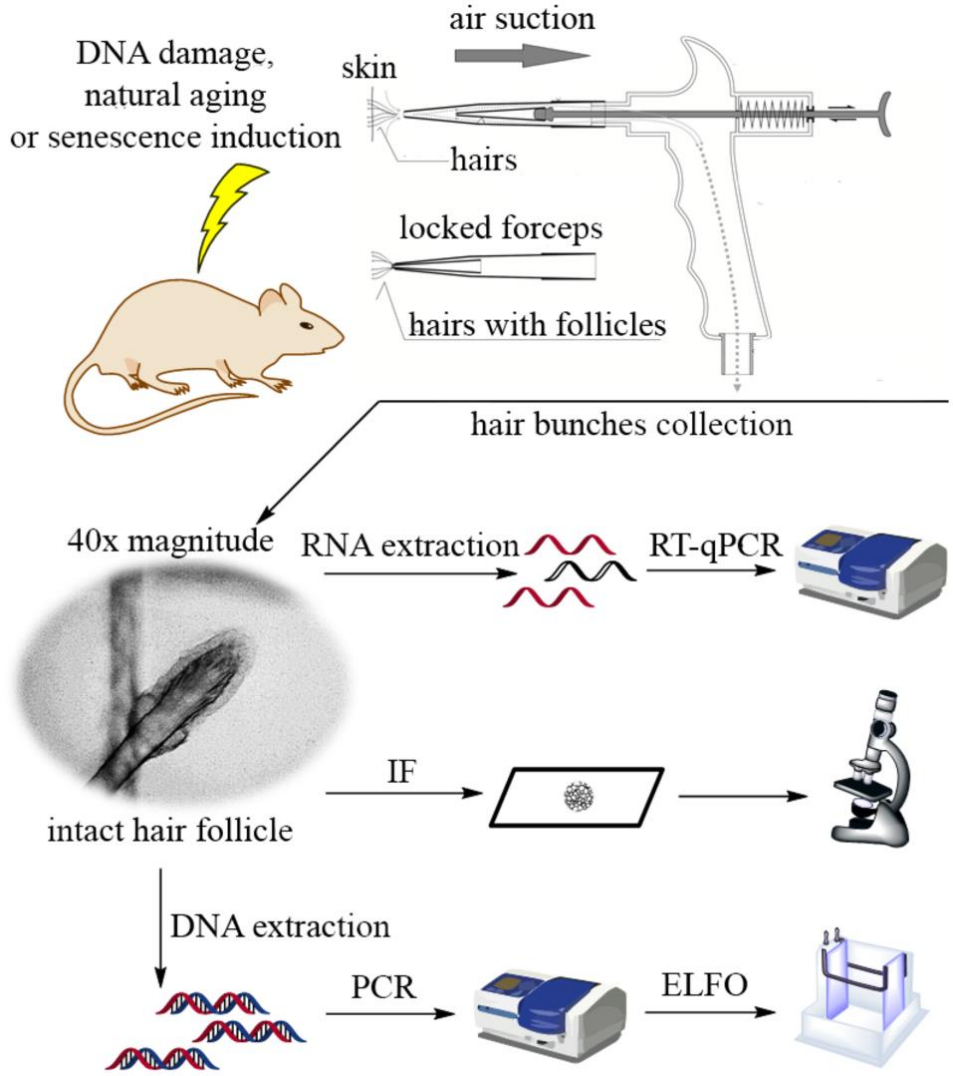
Design of the customized suction-based collector of hair follicle samples and scheme of the experimental workflow in the study.

The device we developed sucks hair from skin by vacuum suction. It uses removable disposable forceps of inert fixation-compatible material; forceps are made from a pair of modified pipette tips of different sizes (one inside the other). The bigger pipette tip is connected to a pistol-like device and covers the smaller tip connected to a piston. The smaller tip clicks into the bigger one by piston push, thereby firmly gripping the sucked hairs. The tip is removed from the pistol by a further push of the piston and stays attached to the skin via the squeezed hair. The forceps with gripped hairs are then manually removed. The removed hairs include follicles composed of multiple live follicular cells (Figure 1). Approximately 200 mice hairs can be removed in a single sample and each follicle contains approximately 50 cells. Inert and disposable forceps allow easy manipulation and immediate processing of the sample by fixation or lysis. The process is fast, easy, and does not involve touching the hair (Supplementary Video 1). Other advantages of the method include noninvasiveness, allowing many repetitive multi-site collections with minimal stress to the experimental animals.

### Pre-processing of murine hairs

**Figure 2:**
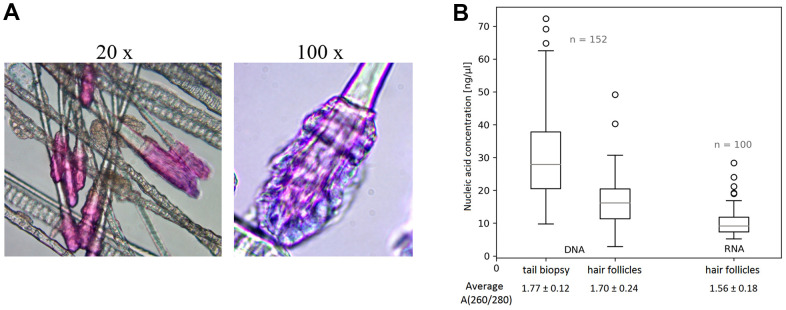
**Murine hair follicles as starting material.** (**A**) Representative images of hairs highlighted with Giemsa – Romanowsky staining (and indicated magnification) showing intact hair follicles obtained by sampling with our vacuum collector. (**B**) Comparison of DNA isolation results from hair follicles obtained with our device and common tail biopsies, and yields of RNA isolated from hair follicles. The quantity and quality of all nucleic acid samples, in 30 μl solutions, were measured using a ND 1000 Spectrophotometer. Data are presented as boxplots showing median, maximum and minimum values, their quartile distribution, interquartile range, and outlier values.

Hair follicles are morphologically intact and can be easily recognized during microscopic evaluation (Figure 2A). Collected hairs can be stored in a dry state or suitable medium, e.g. for nucleic acid extraction. We obtained median yields of 275 and 486 ng of total RNA and DNA per pluck of hairs obtained with our collection device according to analyses with a NanoDrop 1000 Spectrophotometer (Thermo Fisher Scientific). Moreover, the quality of RNA is usually high and suitable for further analyses with a RIN (RNA integrity number) of ca. 8 units according to analysis with an Agilent Bioanalyzer 2100. Thus, we consider DNA and RNA yields and qualities, presented in Figure 2B, sufficient for further experiments. There is no need for any special processing steps such as homogenization for isolating nucleic acids or cutting slices for microscopic analyses, and the material can be collected and processed repeatedly, within short times and in high amounts, from mice.

### Genotyping of collected follicular cells fully replaces tail biopsies

**Figure 3:**
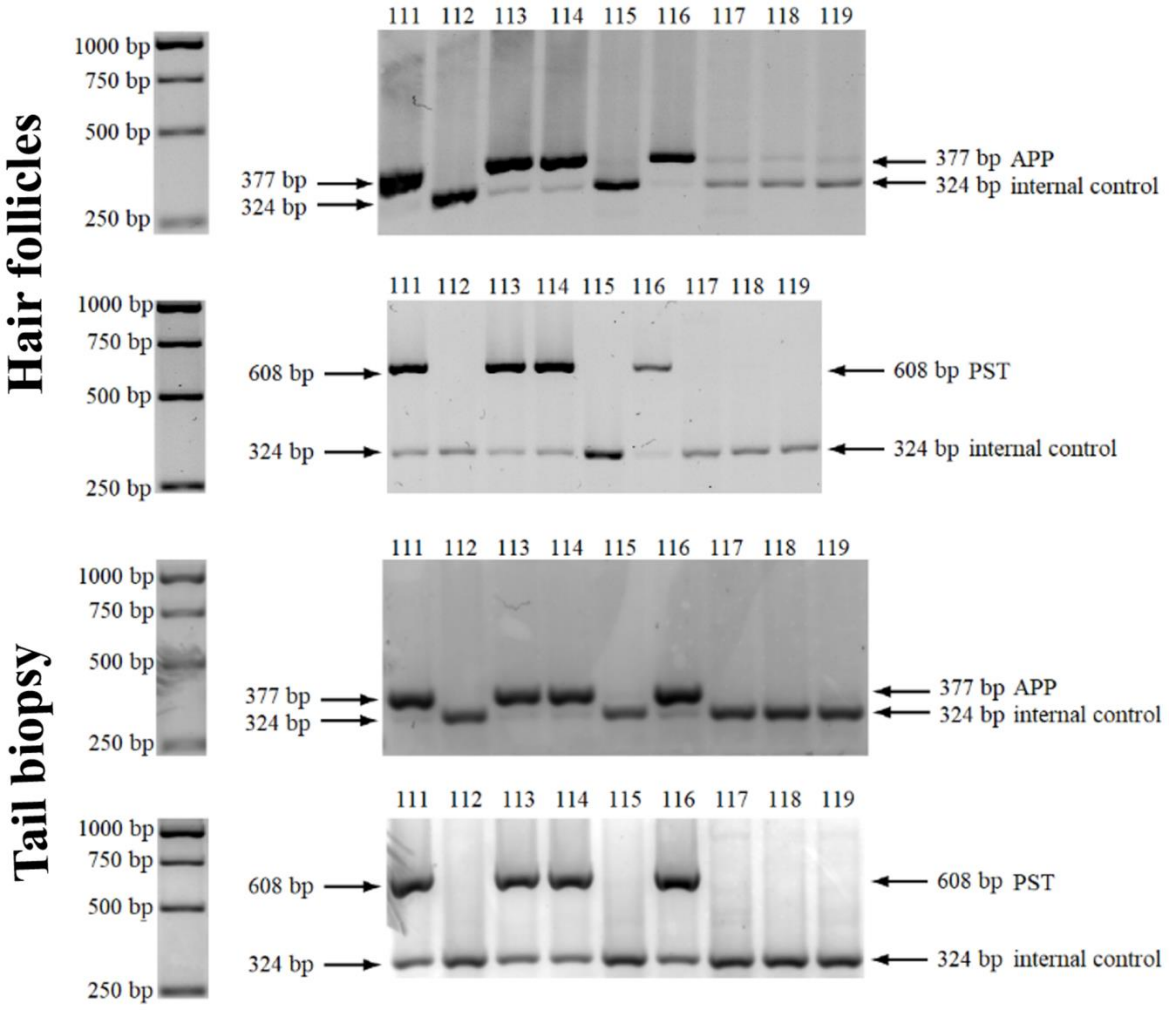
**Genotyping using murine hair follicles.** Results of PCR-agarose gel electrophoresis showing the presence or absence of *APP* and *PST* transgenes in representative samples (from mice 111 – 119), of both biological materials - hair follicles and tail biopsies. A 1 kb GeneRuler DNA ladder is presented on the left side.

Genotyping is one of the most common reasons for biopsy in research and commercial procedures involving genetically modified mice. Usually, a tip of the tail or an ear punch is collected from the examined mice to obtain DNA for analysis. To minimize pain and stress, the ethical standards of many countries require anesthesia before the collection, which complicates and prolongs the procedure. Here we show, that follicles collected by the specialized device described above can fully substitute tail tip biopsies. 5XFAD mice commonly used as a model for Alzheimer's and senescence-related diseases [11, 12], were used for proof of this concept. We compared the genotyping results from 151 tail biopsies and 151 hair samples from the offspring of 5XFAD transgenic males and C57Bl/6 females. For this, we assessed by PCR the presence or absence of two genes carried by the transgenic mice: *APP695* (*APP*, encoding an amyloid-beta [A4] mouse/human chimeric precursor protein) and *PSEN1* (*PST*, encoding human presenilin 1). Results obtained with DNA samples from hair bunches and tail biopsies were fully consistent (presence of both transgenes or absence of both, with no sample showing presence of only one of the transgenes). Thus, hair follicles could apparently fully replace tail biopsy for genotyping, with no loss of accuracy. Results of electrophoretic separation analysis demonstrating the transgenes’ presence or absence are shown in Figure 3.

### Downstream transcriptional DNA damage response in hair follicle cells after gamma irradiation *in-vivo*

**Figure 4:**
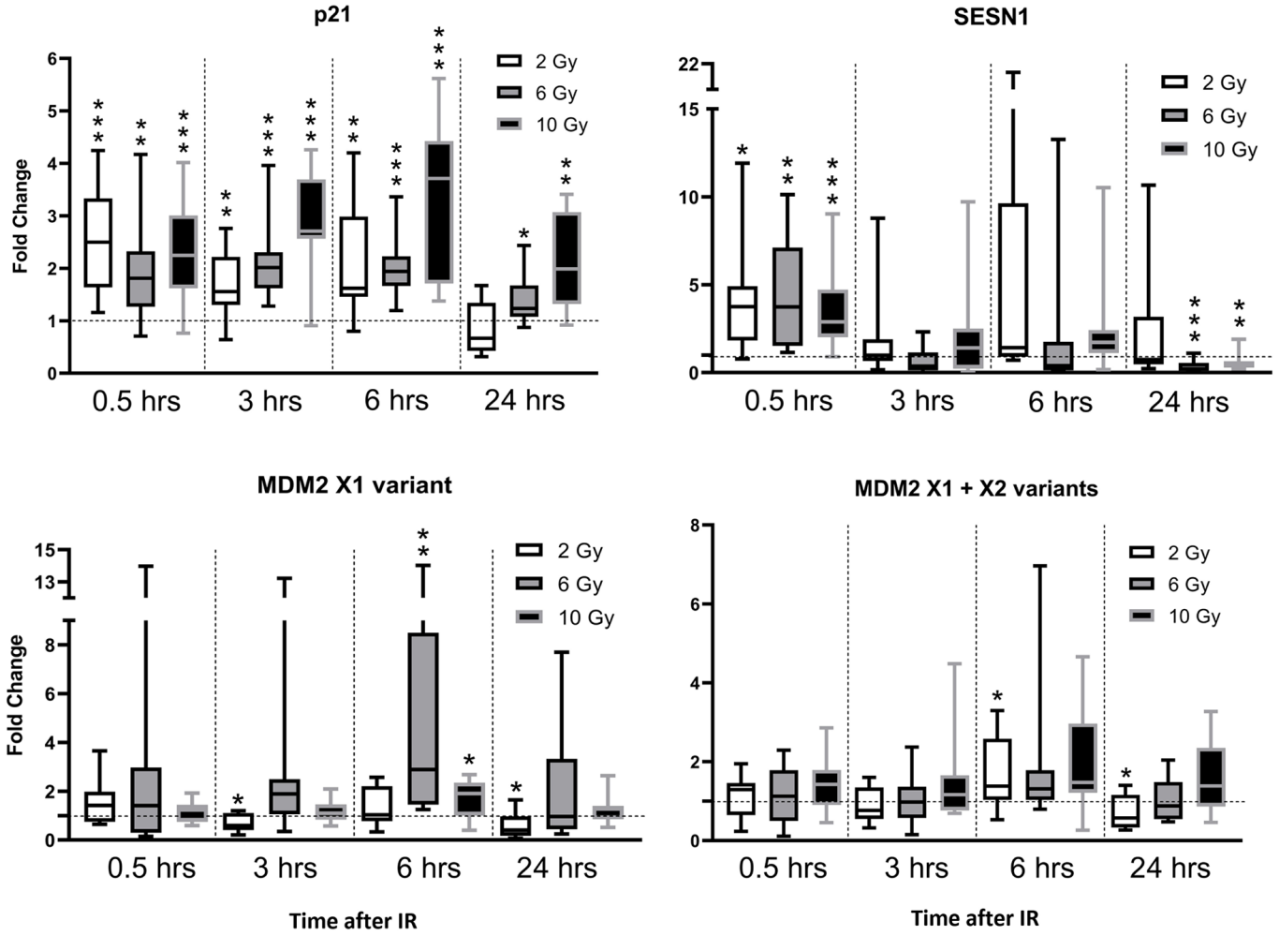
**Gene expression changes (mRNA level) in murine hair follicles after *in vivo* ionizing irradiation.** Data are expressed in boxplot graphs showing median, first quartile data distribution and maximum and minimum values of fold changes relative to expression at time zero (immediately before irradiation) normalized using *HPRT* mRNA levels. Results of paired t-tests of the significance of differences between indicated groups and times (relative to the zero-time point) are also depicted (*p<0.05, **p<0.01, ***p<0.001).

Gamma irradiation, the most common mode of cancer treatment, causes complex responses in exposed cells called the DNA damage response (DDR). The DDR involves multiple transcriptional and posttranscriptional changes which are intensively investigated in cancer studies, and quantitative evaluation of DDR can be used for quick assessments of radiation exposure, radiotherapy monitoring, and identification of radiosensitive individuals [2, 13, 14]. Three commonly used markers (*p21*, *SESN1,* and *MDM2*) in the ATM/CHEK2/p53 pathway, which directly responds to DNA damage, were tested for acute radiation response by RT-qPCR. The stability of their expression in the absence of IR was checked and *p21* showed the least variability between samples obtained on multiple occasions (significance of between-time differences: p = 0.530). In contrast, there were significant between-time differences in *SESN1* expression in irradiated samples and even in the absence of IR (p = 0.025). We also consider *SESN1* an inappropriate marker for monitoring hair follicles after IR because it has very low basal expression (late Ct values or negativity). However, there was significant upregulation of *SESN1* 30 minutes after IR and downregulation after 24 h, especially at the highest doses (Figure 4). In further contrast, *MDM2* showed a much more stable amplification pattern, but very weak dependence on IR. Thus, *p21* provided the most valuable amplification pattern, with the lowest variability and highest potential for distinguishing samples subjected to different IR doses and/or collected at different times. We observed a 2.5 median fold change (FC_m_) 30 min after a 2 Gy dose, followed by a reduction to -1.54 after 24 h (Figure 4). A 6 Gy dose induced a longer response, with FC_m_ = 1.81 at 30 min after radiation, and no observed reduction until the 6 h time-point (FC_m_ = 1.94) and slight reduction to FC_m_ = 1.24 at the 24 h time point. The 10 Gy dose even induced an increase between the 30 min and 6 h time points (FC_m_ = 2.25 and 3.71, respectively). In summary, *p21* showed the most significant increase (p < 0.01) until 6 h after IR at all tested doses (Figure 4), with clear between-dose distinctions at some time points (with higher doses inducing higher FC_m_ values, except at the initial sampling point, 30 min after IR). In summary, our data provide some proof of concept by showing that our device and protocols can detect changes in expression patterns induced by external factors.

### Immunofluorescence (IF) analysis of γ-H2AX lesions after gamma irradiation

**Figure 5:**
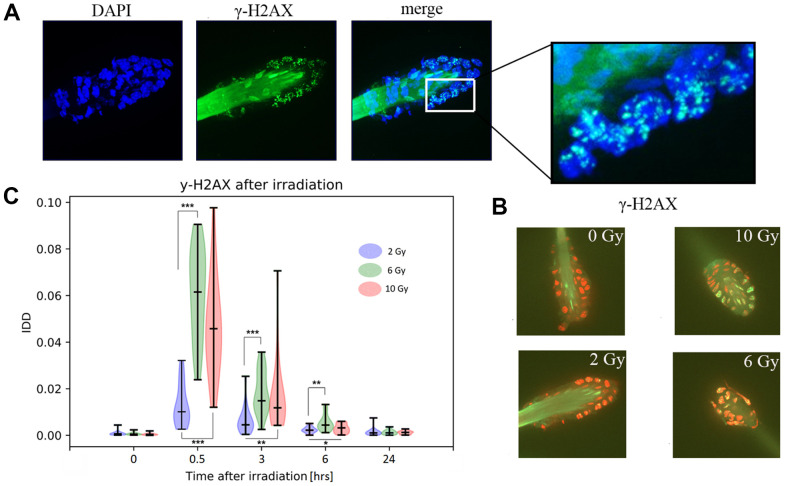
**Immunofluorescent detection of γ-H2AX in murine hair follicles.** (**A**) Representative images of a follicle after ionizing irradiation, extracted as maximum intensity projection from z-stack scanning by confocal spinning disc microscopy. Nuclei stained with DAPI and γ-H2AX foci and hair shaft autofluorescence are visible in the green channel. (**B**) Representative images used in the computational analyses of one z-stack layer, the red color of the nuclei (DNA) was selected artificially to maximize the visibility of green γ-H2AX foci. (**C**) Results of image analyses in terms of IDD (intensity of DNA damage), with each data point representing the area of the γ-H2AX signal related to the area of the nuclei in one scanned hair follicle in z-stack mode. Probability density of the data, maximal and minimal values, medians, and results of a multiple Kruskal-Wallis test (*p<0.05, **p<0.01, ***p<0.001) are showed in violin plots.

In the previously mentioned ATM/CHEK2/p53 pathway and DDR, ATM kinase is directly recruited to DNA damage sites, where it phosphorylates histone H2AX on serine S139, forming γ-H2AX detectable as the immunofluorescent foci pattern in nuclei (Figure 5A - detail). We identified typical intense foci in the nuclei of irradiated hair follicles (Figure 5A). An IDD (intensity of DNA damage) value was calculated, as described in the methods section, for each hair follicle projection as averages from z-stack imaging (Figure 5B). Three to five morphologically intact follicles from each sample were scanned and analyzed. There were no statistically significant differences between measurements from either the same or different mice under defined conditions. Thus, we did not detect any inter-individual variability and there was no distinction between biological and technical replicates in further calculations. The low numbers of subjects in our experiments (n = 5 per condition) may have contributed to this, but they were sufficient for clear quantitative resolution of samples differing in radiation dose and/or time after IR. There was a strong increase in IDD shortly after irradiation, with a gradual reduction during the following 24 h. The IDD was also quantitatively dependent on dose, with clear separation between samples given 2 Gy from those given 6 and 10 Gy doses. Thus, the direct DDR after IR quantitatively differed between doses, but followed similar dynamic patterns under all three doses, in the murine hair follicles (Figure 5C). Not only recognizable foci were detected, but also areas with continuous green γ-H2AX signal, which we attributed to apoptotic lesions, based on previous reports [2] and their general occurrence in samples from mice given the 10 Gy treatment. The 10 Gy treatment also resulted in lower IDD values than the 6 Gy treatment, possibly due to cell damage exceeding thresholds allowing proper quantification, although there was no significant difference between 6 Gy- and 10 Gy-treated samples at any time point. The other between-dose differences in samples collected 30 min, 3 h, and 6 h after IR were significant according to the Kruskal-Wallis non-parametric test with multiple comparisons.

Apart from gamma irradiation, we tested also the possibility to induce DDR after topical application of chemical clastogens. To address such an option, we dissolved DNA damaging agents bleomycin and cis-platin in DMSO and applied them directly to small areas of the skin of tested animals. Collected hair follicles from the exposed areas indeed showed increased γ-H2AX staining in the nuclei compared to the control areas (Supplementary Figure 3). These data confirm that the topical application of various chemical compounds and direct assessment of their effects in follicular cells *in-vivo* is possible which enriches the portfolio of applications of the method significantly.

### Expression patterns of selected markers in mice hair follicular cells reflect their biological age

**Figure 6:**
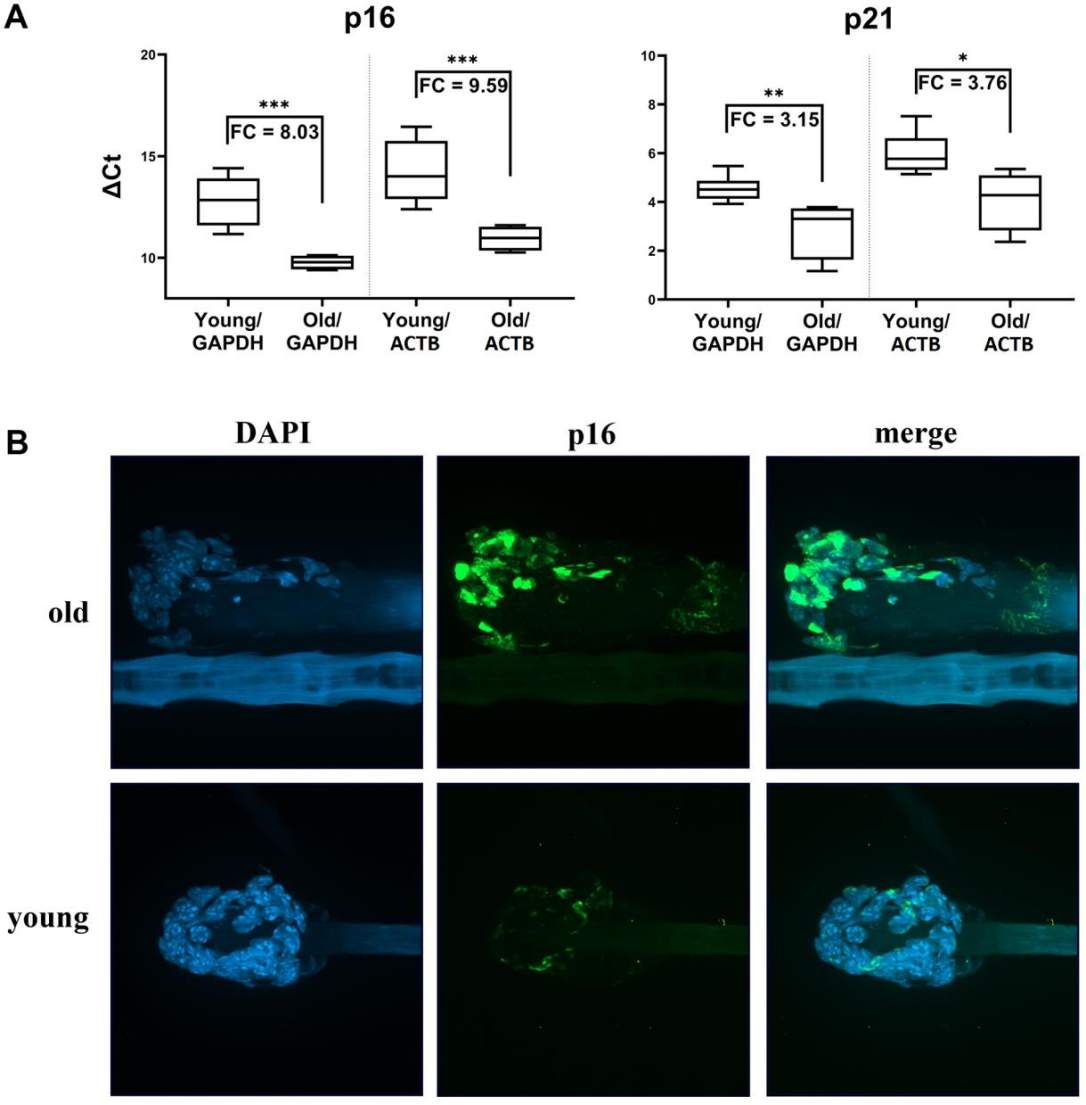
**Markers of senescence analysis in hair follicular cells.** (**A**) *p16* and *p21* gene expression (mRNA level) in hair follicles from young mice (6 months old) and old mice (2.5-3 years old) in boxplot graphs of ΔCt values normalized using *GAPDH* or *ACTB*. Graphs show medians, first quartile data distribution, minimal and maximal points, and fold change values as a specifying detail. Results of a t-test are also shown (*p<0.05, **p<0.01, ***p<0.001). (**B**) Representative images showing the higher p16 protein levels in murine hair follicles of old animals relative to those of young animals. Images were obtained from z-stack scanning with a confocal spinning disc microscope. DAPI-stained nuclei are shown in the blue channel and p16 signals in the green channel. Autofluorescence can be seen in a hair shaft. Magnification 60x objective with oil immersion.

Senescent cells are commonly identified by increases in senescence-associated β-galactosidase (SA-β-gal) activity [15–17], but assays of this activity have multiple limitations [18]. In contrast, *p16* and *p21* have known senescence-associated activities and functions [19–21], and their expression is highly elevated in a wide variety of human and mouse senescent cell lines, so we used them as biomarkers for our age-related senescence measurements [22–24]. qPCR analyses of *p16* and *p21* expression levels using the acquired cDNA samples revealed significant differences in these two senescence biomarkers’ expressions. *Inter alia*, there were strong differences in gene expression between young (6 months old, n=6) and old (2.5-3 years old, n=6) animals (Figure 6A) with p < 0.05, p < 0.01 or p < 0.001 depending on the housekeeping gene used for the data normalization and the target gene. Fold change differences in expression between the young and old animals of 8.03 and 9.58 (p < 0.001) were calculated for the *p16* marker, and 3.15 (p < 0.01) and 3.76 (p < 0.05) for *p21*. Naturally, no significant difference in expression of target senescence markers was detected using animals with the age difference of just a few months (data not shown). To support our qPCR data, we confirmed the changes in p16 expression at the protein level using immunofluorescence assays and the same mice cohort, which showed that cells from the older animals had higher levels of p16 protein (Figure 6B).

### Radiation-induced senescence detection in murine hair follicles

**Figure 7:**
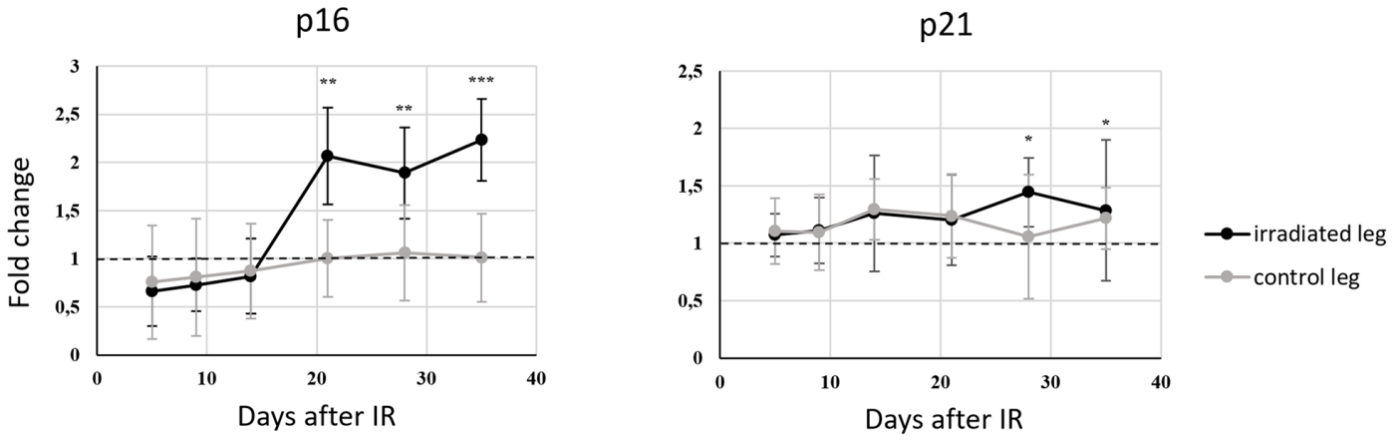
**Changes with time in *p16* and *p21* gene expression (mRNA level) in murine hair follicles after a fractionated dose of 3 x 8 Gy applied *in vivo* to the right hind leg of a mouse, using each animal’s left hind leg as a control.** Data are expressed as fold changes relative to expression at the zero-time point (shortly before irradiation) normalized using *GAPDH* mRNA levels. Means and standard deviations are shown. ΔCt values from certain time points were compared using paired t-tests and relevant time points on curves are marked with asterisks (* p<0.05, ** p<0.01 and***p<0.001).

The number of senescent cells in tissues can also be increased artificially by exposure to various DNA damaging factors. Gamma radiation is a very well-known inducer, and even mildly dosing of cells or animals can generate detectable senescence features [25, 26]. In addition, patients who have undergone anticancer chemo- and/or radiotherapy typically have higher numbers of senescent cells than non-treated counterparts of the same chronological age [27, 28]. We detected clear irradiation-dependent senescence induction in a mouse model, as irradiated soft tissue in the right hind leg acquired more senescent cells (according to expression levels of the *p16* and *p21* markers) than control tissue in the left hind leg. Hair follicles were collected at seven time-points (the first before IR) from the left and right hind legs of 10 mice. Significant elevation in expression of the selected *p16* and *p21* markers was observed in irradiated legs from 21 and 28 days after IR, respectively, relative to levels in samples collected directly before IR. Both markers remained constantly up-regulated until the last sampling time (35 days after IR), clearly indicating that senescence was induced in the irradiated tissue. The quantitative changes were not very strong, the highest observed FC_m_ was 2.26, for the *p16* marker 21 days after IR. However, the changes were significant (p < 0.01) at all of the last three sampling times for *p16* expression. The other marker, *p21*, showed weaker potential for discrimination between senescent and normal tissue in mice using hair follicle samples, with FC_m_ peaking at 1.25, 28 days after IR (p < 0.05). The expression of both markers remained unchanged in hair follicles of the control left legs during the experimental investigations (Figure 7). Those findings prompted the idea of using hair follicle cells as a murine irradiation-induced senescence model for studying senescence markers, aging experiments, and *in vivo* senolytic drug identification.

## DISCUSSION

To meet ethical standards, experiments involving laboratory animals should be designed to obtain maximum information while minimizing stress, and ideally, they should be replaced wherever possible. Unfortunately, they cannot currently be replaced by alternative assays in numerous applications in basic and pre-clinical research. However, we present here a simple method for obtaining biological material in the form of follicular cells from laboratory mice with sufficient quantities and quality for multiple analyses using standard modern molecular biology methods. The material has numerous advantages, including the convenience, non-invasiveness, and repeatability of the sample collection together with its contents of cells of epithelial origin and distal tissue, as well as the ease of exposing follicular tissue to test substances with topical applications (Supplementary Figure 3). The biggest limitations of follicular cell collection in the past were the complex logistics involved in processing the samples. Ordinary tweezers and forceps used in previous setups are extremely impractical with a high risk of cross-contamination, thus this material has been used in relatively few studies. We have circumvented these limitations by using our simple device, which enables a fast, convenient collection of follicular cells. The speed by which the samples can be collected and processed (e.g. by fixation) is among the biggest advantages of our solution as it can be performed within seconds. This fact limits any potential underlying cellular responses and additional DDR caused by cofounding stressing factors related to the withdrawal process [2]. To corroborate the method’s practical utility, we selected three applications. First, we proved that a sample of mouse hairs collected using disposable forceps in less than a minute (Supplementary Video 1) provides enough DNA for analysis by standard genotyping methods developed for tail tip biopsies or ear punches. As millions of laboratory mice are routinely genotyped globally every year this approach represents a major ethical and logistic breakthrough. Compared to such standard methods of biological biopsies (including phlebotomy) the amount of distress to the laboratory mice is relatively low caused mostly by the animal hand-grip as there is no visible pain reaction during the process of hair withdrawal (no twitching and/or squeaking). The absence of pain is supported also by the fact that the mice hair roots are, compared e.g. to humans, embedded relatively shallowly in upper skin layers [4, 10, 29] and very little strength is necessary for the withdrawal process.

Although hair samples have been previously used for that purpose [29–31], our sample collection approach may motivate researchers to use them more routinely and widely.

As we showed, mRNA can also be obtained from the collected samples in sufficient quantity and quality, so we designed another set of experiments in which we monitored changes in the expression of selected genes using qPCR. We targeted genes that respond to DNA damage caused by gamma irradiation and genes whose expression changes during oncogenesis. The DDR is intensively studied in carcinogenesis and cancer treatment research. IR markers often respond differently in different biological materials, so there is no universally optimal marker that consistently provides quantitative responses, especially on the mRNA level. However, acute DDR has been detected accurately and dose-dependently by monitoring the dynamics of *p21* in previous studies [14, 32–34]. We dedicated *p21* as a suitable marker for gene expression measurements in hair follicles. It is also known that responses to IR differ and topically applied drugs can be more meaningfully evaluated using hair follicles than blood samples [2, 6]. Moreover, *p16* mRNA levels differ between samples of young and old human skin [35], which supports our use of its qPCR-based quantification to distinguish follicular cells of young and old mice. The marker has also been applied, for more than 10 years, in analyses of processes associated with senescence caused by G1 arrest, and thus oncogenesis [36], as well as for monitoring tissue degeneration (which can be regarded as premature tissue aging) caused by gamma irradiation or cytotoxic drugs [37]. Irradiation was used as the inducing factor in our artificially triggered senescence experiments, in which we applied fractionated doses of IR based on recent literature [38, 39], clinical experience together with our previous knowledge and optimization. We did not apply a single high IR dose as in previous studies [40] to avoid the formation of post-radiation skin lesions, which did not develop when using fractionated doses. Our approach opens new possibilities for research to find new drugs that can selectively eliminate senescent cells from organisms [41]. Thus, monitoring *p16* and *p21* mRNA levels by this method might replace laborious and less accurate methods, such as SA-β-gal assays in ‘senolytics’ research [42].

We also optimized the immunofluorescent (IF) approach for analyzing the collected follicular cells and thus monitoring selected proteins, including quantitative microscopy-based analysis of the DNA lesion marker γ-H2AX after gamma irradiation (Figure 5). For this, we developed software for routine evaluation of IDD by analysis of γ-H2AX foci in z-stack images of individual hair follicles and investigation of acute DDR after IR, which was made publicly available (https://doi.org/10.6084/m9.figshare.14822643.v1). The temporal dynamics we observed in our samples are consistent with previous results [2, 43] and generally, we proved that murine follicular cells can be used in IF assays of both nuclear and cytoplasmic proteins (Supplementary Figures 1, 2). It might be that the sensitivity of the method will be able to map functional differences between various GMO animals on the cellular level [4, 8]. For example, GMO mice harboring changes in genes involved in DDR could reveal differences in the DNA repair dynamics within the nuclei of follicular cells. Moreover, such tests could be also performed *ex-vivo*, because collected follicular cells are viable [5], without compromising genome integrity [2], and can be kept alive for a significant time allowing various functional tests (Supplementary Figure 4). However, further research is needed to confirm such intriguing potential applications.

Last but not least, the method can be easily adapted for sampling other laboratory mammals, as hairs can be readily collected from rats, rabbits, dogs, monkeys, and even humans by the presented device (Supplementary Figure 4), opening a plethora of future research and biomedical applications.

## MATERIALS AND METHODS

### Ethics statement

All investigations were conducted following the ethical standards stated in the Declaration of Helsinki and other relevant national and international guidelines, with approval of the author's institutional review board.

### Mouse hair collection

We collected each sample using an extraction device with modified pipette tips (described in patents EP2928382A1 and US 20150297253). We then placed the tips with the samples directly into 500 μl portions of RNAlater (Qiagen, Hilden, Germany) for RT-qPCR analysis or 500 μl of 10% neutral buffered formalin solution (Sigma-Aldrich, St. Louis, MO, USA) for immunofluorescence assays.

### Human hair collection and *ex-vivo* irradiation

Hair follicles were collected from surface disinfected human calves and immediately put into the Dulbecco’s modified Eagle’s medium containing 4.5 g/l glucose (Biosera, Nuaille, France), supplemented with 10% fetal bovine serum (Gibco/Thermo Fisher Scientific, Grand Island, NY, USA) and 1% penicillin-streptomycin (Sigma-Aldrich). Samples were stored in the cell culture medium under normal cultivation conditions (5% CO2, 37° C, 100% humidity) during the necessary incubation steps until the fixation for immunofluorescence assays. Human hair follicle samples were irradiated on Petri dishes with DMEM culture medium using X-ray RS225M Research Cabinet (Xstrahl, Suwanee, GA, USA) with a 2 Gy dose. Samples were fixed 30 min after irradiation and processed in the same way as described below in the „Immunofluorescence and image acquisition” chapter.

### Irradiation

Thirty C57Bl/6 wild-type mice (Envigo, Huntingdon, UK) were used in the first part of the study, 10 mice for each dose of ionizing radiation. An RS225M Research Cabinet (Xstrahl, Suwanee, GA, USA) was used to generate X-rays in all experiments. Each mouse was anesthetized with isoflurane (FORANE inhalation solution, Aesica, Hemel Hempstead, UK) before and during the application of the radiation. Only the right hind leg and adjacent parts of the body were irradiated, while the rest was covered by lead armor. Additionally, 10 mice Balb/cOlaHsd (Envigo, The Netherlands) were irradiated to induce senescence in muscle tissue with a fractionated dose of 3 x 8 Gy.

### Topical application of chemical clastogens

HsdWin:NMRI mice (Envigo, Huntingdon, UK) females (3 animals per group) were treated with two clastogens (bleomycin or cis-platin) topically. Both compounds were dissolved in DMSO. We applied 16,6 μl of bleomycin solution (5 μg/ml) and 30 μl of cis-platin solution (0,5 mg/ml). Each mouse was treated on the right body side by gentle rubbing of the compound into the skin. The treated spot had approximately 1,5 cm in diameter, each treated spot was marked. Bleomycin-treated animals were incubated in their homecages for 1 hour and animals treated with cisplatin were incubated for 5 hours. Then we collected hair samples (treated samples from the marked spot, control samples from the left body side). All samples underwent IF staining for γ-H2AX detection.

### DNA isolation and genotyping

We genotyped the offspring (151 animals) of 5XFAD transgenic males (B6Cg-TgTg(APPSwFlLon, PSEN1*M146L*L286V)6799Vas/Mmjax) obtained from The Jackson Laboratory and C57Bl/6 JOIaHsd females from Envigo (Huntingdon, UK) as genetic background. Genomic DNA was isolated from both mouse hair follicles and mouse tail biopsies with a Cobas**®** DNA Sample Preparation Kit (Roche, Basel, Switzerland) according to the manufacturer’s instructions. The presence of APP and PST transgenes was confirmed by optimized PCR, the reaction mixture contained 2 μl of template sample, 2 μl of 10 μM APP or PST primer mix, 2 μl of 10 μM internal control primer mix, 5 μl 5x GC buffer, 0.5 μl 10 mM deoxyribonucleotide triphosphates (dNTPs), 0.75 μl DMSO, 0.25 μl Phusion^®^ High-Fidelity DNA Polymerase) and 12,5 μl nuclease-free water (reagents from New England Biolabs, Ipswich, MA, USA). Primers are listed in Supplementary Table 1. Reactions were performed using the following program: 98° C for 30 s, followed by 30 amplification cycles of 98° C for 10 s, 63° C for 30 s, 72° C for 10 s, then 72° C for 5 min and cooling afterward. PCR products were visualized and checked using 1% agarose/TBE gels and an Odyssey Fc electrophoresis system (LI-COR, Lincoln, NE, USA). The presence or absence of target genes was determined and animals were categorized as GMO-positive (with both transgenes present) or GMO-negative (lacking both genes). Each sample had to contain a band for internal control (bp).

### RNA extraction and reverse transcription

Total RNA was extracted from hair stacks lysed in 700 μl of QIAzol Lysis Reagent and processed immediately using a miRNeasy Mini Kit (Qiagen, Hilden, Germany) according to the manufacturer’s recommended protocol, except the need to discard hair samples before solutions were transferred into columns. Finally, RNA from each sample was eluted into 30 μl RNase-free water. RNA concentration and quality were assessed by a Nanodrop ND 1000 Spectrophotometer (ThermoScientific, Wilmington, DE, USA). To obtain cDNA, total RNA was used for reverse transcription using 18.9 μl of each sample, mixed with 0.3 μg of Random Primers (Promega, Madison, WI, USA) to a final volume of 19.5 μl and incubated at 70° C for 5 min. Then, samples were cooled for 1 min. A solution containing 6 μl of RevertAid 5x RT buffer (Fermentas, Vilnius, Lithuania), 3 μl of 10 mM deoxyribonucleotide triphosphates (dNTPs), and 0.75 μl of 40 U/μl RNAsin ribonuclease inhibitor (Promega) was added to each sample. The mixed solutions were incubated for 5 min at room temperature. In the last step, 150 U (0.75 μl) of RevertAid Moloney Murine Leukemia Virus reverse transcriptase (Fermentas) was added to each sample. The final 30 μl mixtures were incubated at room temperature for 10 min, 42° C for 60 min, and 70° C for 10 min. The resulting samples of cDNA were stored at -20° C. RNA for the subsequent aging experiment was obtained from mouse hair follicles of the mouse strain used for genotyping.

### Quantitative PCR (qPCR)

For quantification of *p21*, *SENS1*, *MDM2* expression, as studied markers, and expression of *HPRT* as a housekeeping gene for relative quantification in the radiation experiment, real-time PCR was performed using a LightCycler 480 instrument (Roche). SYBR Green intercalation chemistry was used to detect the fluorescence signal and supplied software was used for Ct data generation and Tm analyses. An 18 μl volume solution of each sample was mixed with 12.43 μl DEPC-treated water (Ambion, Austin, TX, USA), 2 μl 10X Thermo-Start PCR Buffer, 1.6 μl of 25 mM MgCl_2,_ and 0.2 μl of 5 U/μl Thermo-Start Taq DNA Polymerase (Thermo Fisher Scientific, Waltham, MA, USA). A 0.61 μl portion of SYBR^®^ Green I nucleic acid gel stain (10 000 x in DMSO) diluted in 10 mM Tris-HCl buffer pH 8 (Sigma-Aldrich) was added to each mixture, followed by 0.16 μl of 25 mM dNTPs (Promega) and 1 μl of a solution of specific primers for the selected marker and species. Specific oligonucleotides were custom synthesized by Generi Biotech (Hradec Králové, Czech Republic) and are presented in Supplementary Table 1. Reactions were performed in doublets using 2 μl cDNA. Touch-down programs were chosen for murine samples with the following cycling parameters: 95° C for 10 min, then 95° C for 15 s and 67° C for 20 s with a target temperature of 62° C and 1° C steps for the first touchdown, and 95° C for 15 s and 60° C for 20 s with a target temperature of 58° C and 2° C steps for the second touchdown. Fluorescence signals were acquired during 50 cycles of 95° C for 15 s and 54° C (60° C for *MDM2*) for 45 s. For qPCR analyses of senescence marker (*p16* or *p21*) expression and housekeeping genes (*GAPDH* and *ACTB*), 2 μl of cDNA of each sample was used within reaction mixtures further containing 10 μl LightCycler 480 probes Master 2x conc. (Roche), 7 μl LightCycler 480 Probes Master H_2_O PCR grade (Roche) and 1 μl FAM-MGB Taqman probe (ThermoFisher Scientific). Reactions were performed using the following program: 95° C for 10 min, followed by 50 amplification cycles of 95° C for 15 s and 60° C for 60 s.

### Immunofluorescence and image acquisition

Murine hair follicles for IF assays were obtained from HsdWin:NMRI mice (Envigo, UK). All samples were fixed in 10% neutral buffered formalin (Sigma-Aldrich) for 20 min and permeabilized with 0.5%TritonX-100 (Carl Roth GmbH + Co. KG, Karlsruhe, Germany) in phosphate-buffered saline (PBS) for 10 min. After every step, samples were washed in PBS (2 x 2 min). Then they were blocked in PBS containing 1% bovine serum albumin (Sigma-Aldrich) for 20 min. The blocking solution was also used to dilute the antibodies. Samples were incubated in primary antibody solution overnight at 4° C. After washing with PBS, samples were incubated in secondary antibody solution for one hour. The primary and secondary antibodies are listed in Supplementary Tables 2, 3. Samples were washed in PBS and mounted using Vectashield antifade medium with DAPI (Vector Laboratories, Burlingame, CA, USA). Images of mouse hair follicles were obtained using an Axio Observer.Z1/Cell Observer Spinning Disc microscopic system (Zeiss, Oberkochen, Germany) with a 63x oil objective. All images were processed using ZEN Blue Image processing software (Zeiss).

### Giemsa - Romanowsky staining

To investigate the mouse hair follicle cells’ general morphology, we used classical Giemsa-Romanowsky staining, as follows. Immediately after collecting samples of murine hairs in collection tips they were incubated in 4% methanol solution for 15 min, washed three times for 2 min in PBS, then soaked for 30 seconds in May - Grünwald solution (Penta, Prague, Czech Republic) and subsequently rinsed in distilled water for 2 min, gently dried and finally incubated in 10x diluted Giemsa-Romanowsky solution (Penta). The rest of the staining solution was discarded by washing the samples in distilled water for 2 min. Hair endings with follicles were cut and placed between a microscopic glass and coverslip in a water drop. Images were obtained at 20x or 100x magnification with a transmission light microscope (Zeiss, Primo Vert).

### Data processing and statistical analyses

Matlab R2017a and Microsoft Excel were used for all data processing analyses and visualizations. Fold changes in gene expression were calculated from qPCR data using the ΔΔCt method, with averages of raw technical measurements as the inputs. The Kruskal-Wallis test was used for irradiation-dependence verification of the results at zero doses. T-tests were used to investigate differences between irradiated and non-irradiated samples at each time point. T-tests were also used to generate p-values describing the significance of differences between old and young animals and between variables measured at the zero-time point and all other time points in both irradiated and control hind legs in the radiation-induced senescence experiment. In γ-H2AX analyses, green dots and areas were sought in z-stack images (in tiff format) of DAPI-stained tissues. Two median filters were used for automatic identification of the foci and suppression of background signals. The intensity of DNA damage (IDD) was calculated as shares of the signals and DAPI area, where one data point represents the average value from z-stack images from one hair follicle. The software with example 6 Gy data set is publically available on the Figshare platform at the following link: https://doi.org/10.6084/m9.figshare.14822643.v1. The Kruskal-Wallis test was used to test the null hypothesis that there was no treatment- or sample-related differences in technical measurements.

## Supplementary Material

Supplementary Figures

Supplementary Tables

Supplementary Video 1
