# Supplementary material for: An efficient, non-invasive approach for in-vivo sampling of hair follicles: design and applications in monitoring DNA damage and aging
Source: Aging (Albany NY). 2021 Dec 6;13(23):25004–24. doi: 10.18632/aging.203744 (PMC8714131; doi:10.18632/aging.203744)
Supplement: Supplementary Figures [file aging-13-203744-s001.pdf]

SUPPLEMENTARY FIGURES

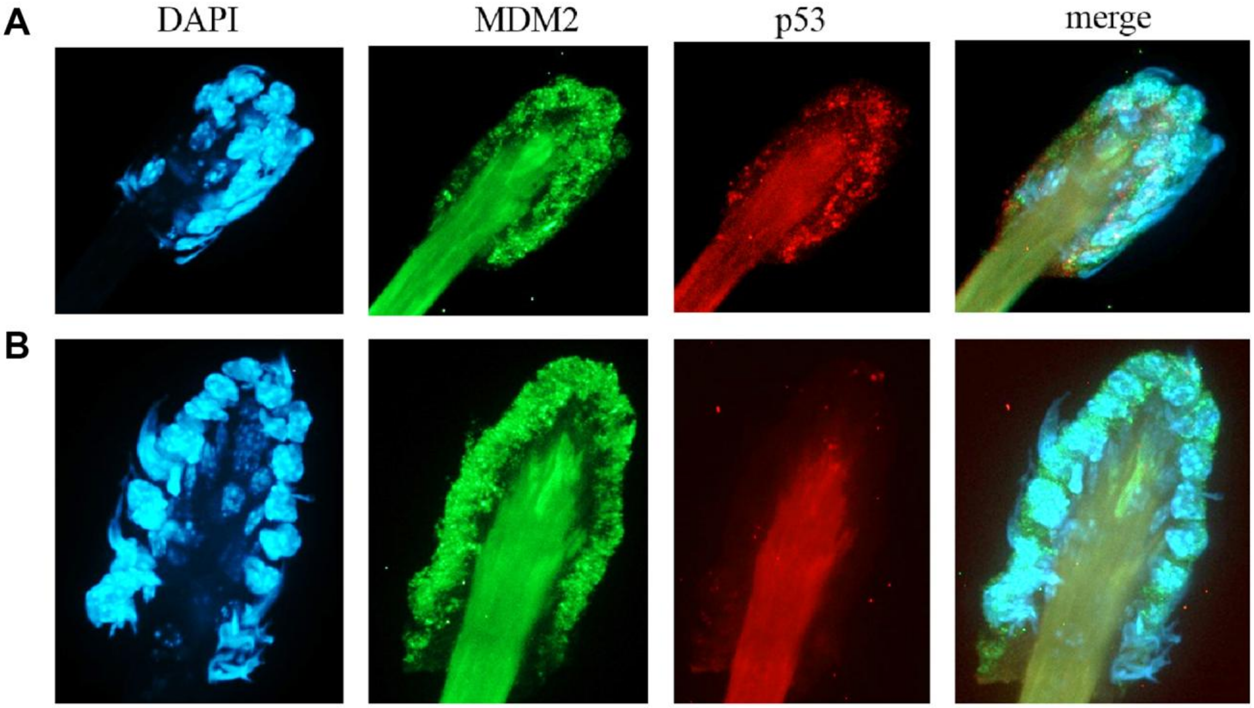

Supplementary Figure 1. Immunofluorescent detection of MDM2 and p53 proteins in murine hair follicles. (**A**) Representative images of a hair follicle as maximum intensity projections from z-stack scanning with a confocal spinning disc microscope. Nuclei are stained with DAPI, green and red autofluorescence is visible in a hair shaft. (**B**) Representative images for autofluorescence estimation in the red channel. No primary antibody for p53 was used to show the difference between autofluorescence and real signals in the red channel. Magnification 60x objective with oil immersion.

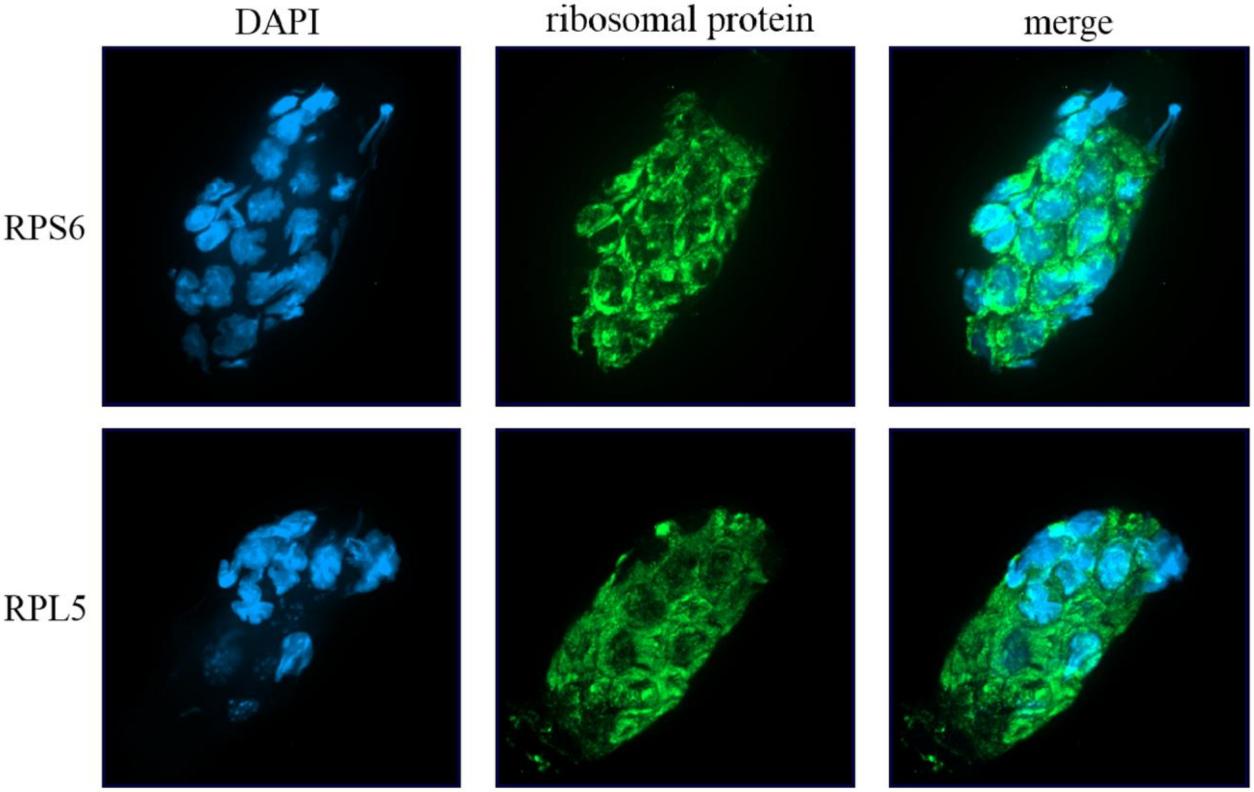

Supplementary Figure 2. Example of immunofluorescent detection of cytoplasmic proteins - ribosomal proteins RPS6 and RPL5 in murine hair follicles. Nuclei are stained with Hoechst 33342 and shown in the blue channel, ribosomal proteins are shown in the green channel. Representative images were obtained from z-stack scanning with a confocal spinning disc microscope. Magnification 60x objective with oil immersion.

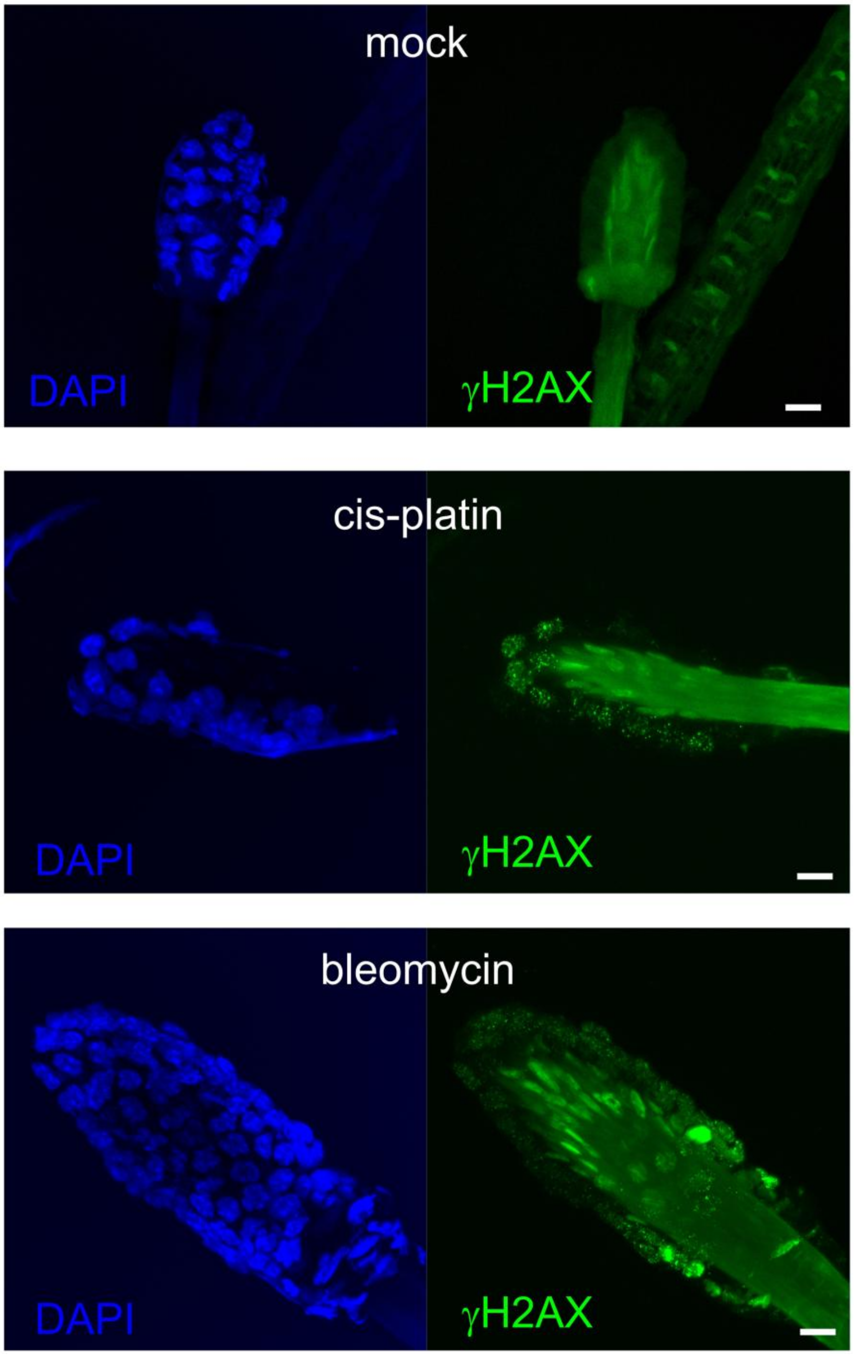

Supplementary Figure 3. Immunofluorescent detection of γ-H2AX in murine hair follicles after the treatment with topically applied clastogens (bleomycin and cisplatin).

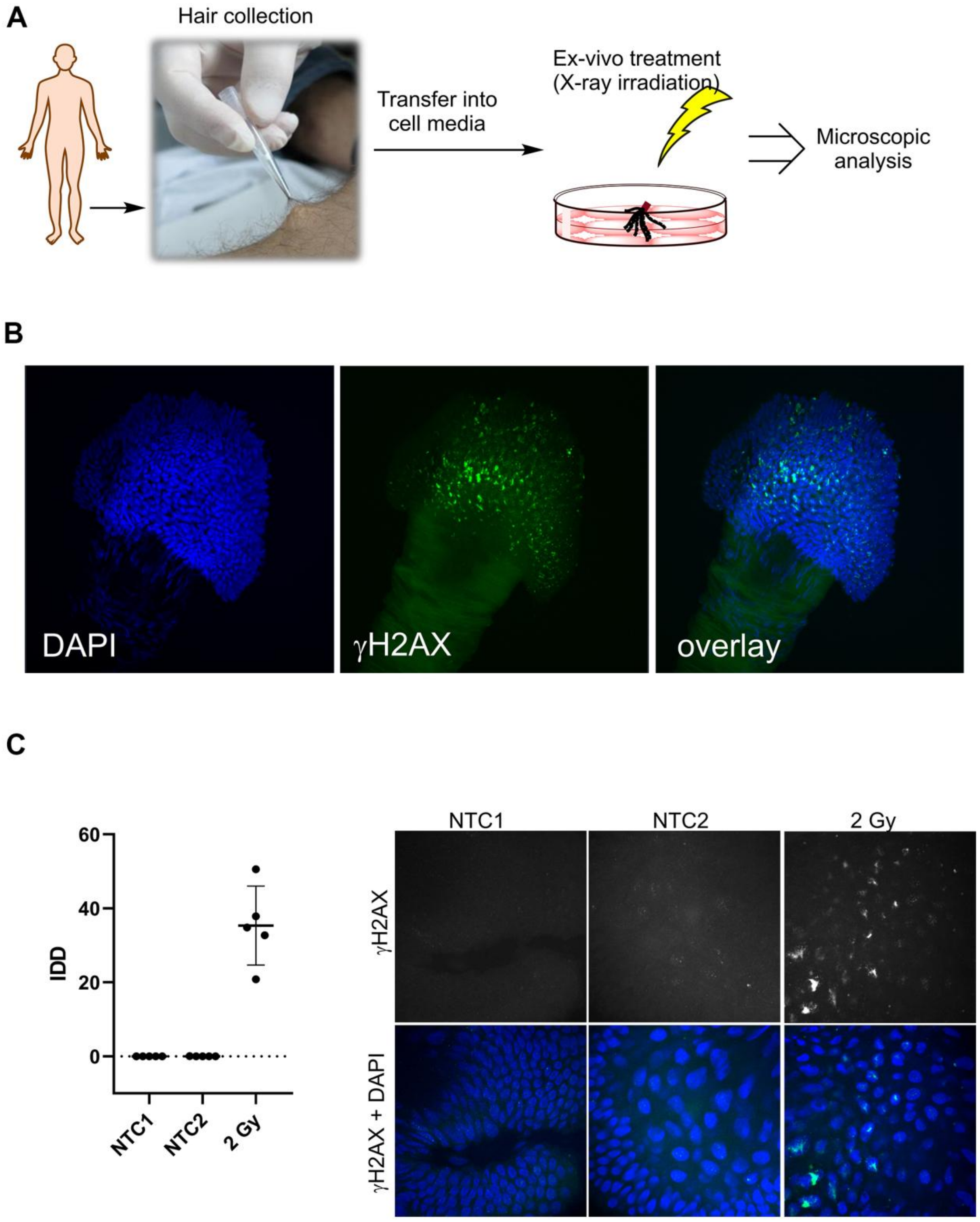

Supplementary Figure 4. Immunofluorescent detection of γ-H2AX in human hair follicles. (**A**) Scheme of the experimental workflow. (**B**) Representative image of human hair follicle *ex-vivo* irradiated by 2 Gy of ionizing irradiation. Nuclei stained with DAPI (blue), γ-H2AX (green). Hair shaft autofluorescence is also visible in the green channel. (**C**) Results of image analyses in terms of IDD (intensity of DNA damage), with each data point representing the area of the γ-H2AX signal related to the area of the nuclei in one scanned hair follicle in z-stack mode and representative images used in the computational analyses of one z-stack layer. NTC1 and 2 are the non-irradiated controls. NTC1 was fixed immediately after the collection and NTC2 was incubated for 30 min in cell media, as well as the 2 Gy samples after irradiation.
