# Supplementary material for: An efficient, non-invasive approach for in-vivo sampling of hair follicles: design and applications in monitoring DNA damage and aging
Source: Aging (Albany NY). 2021 Dec 6;13(23):25004–24. doi: 10.18632/aging.203744 (PMC8714131; doi:10.18632/aging.203744)
Supplement: Supplementary Tables [file aging-13-203744-s002.pdf]

SUPPLEMENTARY TABLES

Supplementary Table 1. Primers for PCR.

Type	Gene	Forward primer 5' - 3'	Reverse primer 5' - 3'	Amplicon
mRNA	*HPRT*	GGACAGGACTGAAAGACTTG	TAATCCAGCAGGTCAGCAAA	114 bps
mRNA	*p21*	GCAAGAGAAAACCCTGAAGTG	CACACAGAGTGAGGGCTAAG	100 bps
mRNA	*SESN1*	CCATAGGCCTTGGCTGATTA	TCCACACTGTGATTGCCATT	246 bps
mRNA	*MDM2 X1*	TGCAAGCACCTCACAGATTC	ACACAATGTGCTGCTGCTTC	188 bps
	MDM2 X1+X2	ATGAATCCTCCCCTTCCATC	CTGTCAGCTTTTTGCCATCA	169 bps
DNA	*APP*	AGGACTGACCACTCGACCAG	CGGGGGTCTAGTTCTGCAT	377 bps
DNA	*PST*	AATAGAGAACGGCAGGAGCA	GCCATGAGGGCACTAATCAT	608 bps
DNA	Internal control	CTAGGCCACAGAATTGAAAGATCT	GTAGGTGGAAATTCTAGCATCATCC	324 bps

Two pairs of primers were designed for MDM2. One (MDM2 X1) only covers transcript variant 1 (NM_010786.4) and the other (MDM2 X1+X2) covers both known variants (NM_001288586.2 as well as NM_010786.4). Amplicon lengths for each marker were verified using an Agilent Bioanalyzer 2100 and Agilent DNA 1000 Kit. Primers for both transgenes (APP and PST) and internal controls were designed and their sequences were provided by the Jackson Laboratory.

Supplementary Table 2. Primary antibodies.

Antigen	Species	Company	Catalog no.	Dilution	Application
p16	Mouse	Santa Cruz Biotechnology	sc-1661	1:100	IF
H2AX	Rabbit	Cell Signaling	#9718	1:400	IF
MDM2	Mouse	Abcam	ab16895	1:200	IF
p53	Rabbit	Santa Cruz Biotechnology	Sc-6243	1:100	IF
RPS6	Rabbit	Cell Signaling	#2217	1:100	IF
RPL5	Rabbit	Cell Signaling	#14568	1:100	IF

Supplementary Table 3. Secondary antibodies.

Host	Recognizes	Fluorophore	Company	Catalog no.	Dilution	Application
Goat	Mouse IgG	Alexa Fluor 488	ThermoFisher	A-1101	1:1000	IF
Goat	Rabbit IgG	Alexa Fluor 488	ThermoFisher	A-11034	1:1000	IF
Goat	Rabbit IgG	Alexa Fluor 488	Abcam	ab150077	1:1000	IF
